# Effects of Climatic Change on the Potential Distribution of *Lycoriella* Species (Diptera: Sciaridae) of Economic Importance

**DOI:** 10.3390/insects12090831

**Published:** 2021-09-15

**Authors:** Roberta Marques, Juliano Lessa Pinto Duarte, Adriane da Fonseca Duarte, Rodrigo Ferreira Krüger, Uemmerson Silva da Cunha, Luis Osorio-Olvera, Rusby G. Contreras-Díaz, Daniel Jiménez-García

**Affiliations:** 1 Departamento de Saúde Coletiva, Faculdade de Ciências Médicas, Universidade Estadual de Campinas, Campinas 13083-970, Brazil; roberta.marques@viep.com.mx; 2 Laboratório de Ecologia de Parasitos e Vetores, Departamento de Microbiologia e Parasitologia, Universidade Federal de Pelotas, Pelotas 96010-610, Brazil; rfkruger@gmail.com; 3 Laboratorio de Biodiversidad, Centro de Agroecología y Ambiente, Instituto de Ciencias de la Benemérita, Universidad Autónoma de Puebla, Puebla 72410, Mexico; 4 Departamento de Fitossanidade, Faculdade de Agronomia Eliseu Maciel, Campus Capão do Leão, Universidade Federal de Pelotas—UFPel, Capão do Leão 96160-000, Brazil; adriane.faem@hotmail.com (A.d.F.D.); uscunha@yahoo.com.br (U.S.d.C.); 5 Departamento de Ecología de la Biodiversidad, Instituto de Ecología, Universidad Nacional Autónoma de México, Ciudad de México 04510, Mexico; luis.osorio@iecologia.unam.mx; 6 Departamento de Matemáticas, Facultad de Ciencias, Universidad Nacional Autónoma de México, Ciudad de México 04510, Mexico; rusby.contreras.diaz@gmail.com; 7 Posgrado en Ciencias Biológicas, Unidad de Posgrado, Universidad Nacional Autónoma de México, Ciudad de México 04510, Mexico

**Keywords:** greenhouse, environmental suitability, mushroom pest, black fungus gnats

## Abstract

**Simple Summary:**

Here, we describe climate change effects on biodiversity, mainly in pest species related to greenhouse production. We used statistical and theoretical methods to describe crops’ vulnerability under worldwide climate change. Some insects (flies) generate economic damage in mushroom, strawberry, and nursery production. We determined potential risk areas for the invasion of three fly species under different climate change scenarios in 2050. Range expansion was determined in the Northern Hemisphere; however, some regions in South America, Africa, and Australia had increases and potentially invasive areas. Our results provide information for farmers, researchers, and politicians for decision-making around production to reduce possible damage caused by pests.

**Abstract:**

*Lycoriella* species (Sciaridae) are responsible for significant economic losses in greenhouse production (e.g., mushrooms, strawberries, and nurseries). The current distributions of species in the genus are restricted to cold-climate countries. Three species of *Lycoriella* are of particular economic concern in view of their ability to invade areas in countries across the Northern Hemisphere. We used ecological niche models to determine the potential for range expansion under future climate change scenarios (RCP 4.5 and RCP 8.5) in the distribution of these three species of *Lycoriella*. Stable environmental suitability under climate change was a dominant theme in these species; however, potential range increases were noted in key countries (e.g., USA, Brazil, and China). Our results illustrate the potential for range expansion in these species in the Southern Hemisphere, including some of the highest greenhouse production areas in the world.

## 1. Introduction

Sciaridae (Insecta, Diptera), known as black fungus gnats, comprise more than 2600 species worldwide, most of which are harmless to human activities [1]. Although most of the species have phyto-saprophagous larvae, 10 known species have larvae that may feed on living tissue, thus damaging roots or mining stems and leaves of economically important crops and ornamental plants, which can lead to significant economic losses [2,3,4,5].

Mushroom crops can be severely affected by sciarids. Sciarid larvae can feed on the developing mycelium inside the substrate and destroy sporophore primordia. Mature mushrooms may also be damaged by larvae tunneling into the tissue, which leads to product depreciation. Severe larval infestations may even destroy the sporophores, causing severe economic losses to producers [6].

Since 1978, worldwide production of cultivated edible fungi has increased around 30-fold and is expected to increase further in coming years [7]. Mushrooms represented a global market of USD 63 B in 2013 [8]. According to the USDA, the value of mushroom sales for 2019–2020 in the USA was USD 1.15 B, up 3% from the previous season [9]. Among the mushrooms produced, *Agaricus bisporus* is the most important, according to the Economics, Statistics and Market Information System. In 2020–2021, the area under production consisted of 12,470 m^2^, 56.5% of which is in the state Pennsylvania [9]. 

The mushroom industry has suffered major economic losses due to sciarid larvae in Australia, USA, Russia, United Kingdom, and South Korea [10,11]. Three sciarid species of the genus *Lycoriella* Frey, 1942 (*L. agraria*, *L. ingenua*, and *L. sativae*) are particularly harmful to cultivated mushroom crops, and are considered to rank among the most important pests of cultivated mushrooms throughout the world [4,10]. In countries like the United States and England, *L. ingenua* and *L. sativae* are the most serious pests in mushroom crops [12], as well in Europe [10]. In Korea, *L. ingenua* is considered to be the most economically important [11]. Given their small size, sciarid larvae can be inadvertently transported to new areas by human activities. Infested potting mix, soilless media, commercial plant substrate, and rooted plant plugs have been shown to act as pathways for sciarid movement [13]. From 1950 onwards, globalization promoted the transport of these invasive species [14]. In this sense, studies of their ecology, environmental requirements, and climatic change impacts for the establishment of invasive populations are needed.

Ecological niche modeling (ENM) is used to evaluate relationships between environmental conditions and species’ abundance and occurrence [15]. Understanding the potential distributions of species represents an important opportunity for pest control and mitigation of possible invaders e.g., [16,17,18]. Considering that the three *Lycoriella* species are economically important and are invasive species [10,19], niche modeling allows researchers to identify areas not currently occupied by them; if dispersal is possible or facilitated, these areas can be invaded and populations established in these regions [15]. For these reasons, we used ENM to identify new regions of potential risk of invasion by three *Lycoriella* species with pest status in mushroom production, under current and future climate conditions (2050) for two greenhouse gas emissions scenarios.

## 2. Materials and Methods

### 2.1. Occurrence Data

Occurrence data for *Lycoriella* species were obtained from published papers available in bibliographic databases (Google Scholar, Web of Science, Scopus; Supplementary Information), and from SpeciesLink (http://splink.cria.org.br/ (accessed on 13 January 2020)) and GBIF (http://www.gbif.org (accessed on 15 November 2020)). We gathered all data from 1950–2018 for synonyms [3] including *L. agraria* [20] and its synonym *Sciara multiseta* [21], *L. ingenua* [22] and its synonym *S. pauciseta* [23] and *L. sativae* [24], and its synonyms *L. auripila* [25] and *L. castanescens* [26]. Occurrence data lacking geographic coordinates were georeferenced in Google Earth (2015; https://earth.google.com/web/ (accessed on 15 February 2020)). We excluded records lacking the exact location or with high geographic uncertainty (e.g., name of the country as a collection site).

We assembled the occurrence data for each *Lycoriella* species at world level, and performed a geographic spatial thinning such that no thick points were closer than 5 km using the spThin R package [27]. As such, we used 43 *L. agraria* occurrences, 118 *L. ingenua* occurrences, and 136 *L. sativae* occurrences. Finally, the data were split randomly into two subsets: 50% for model training and 50% for model testing (Figures S1–S3).

### 2.2. Environmental Variables

The bioclimatic variables used here to summarize climatic variation were from WorldClim version 1.4 [28]; we excluded four variables (bio 8, bio 9, bio 18, bio 19) that present spatial artefacts [29]. We summarized future conditions via 22 general circulation models (GCMs, Table S1) for 2050 available from Climate Change, Agriculture and Food Security [30]. Two scenarios for greenhouse gas emissions (RCP 4.5 and RCP 8.5) were used to explore variation in possible trajectories for future emissions. The climate variables were used at a spatial resolution of 2.5 min (~5 km^2^). We used Pearson’s correlations across each of the calibration areas for each species, removing one from each pair of variables with a correlation ≥0.80. The remaining non-correlated variables were grouped into all possible sets of ≥2 variables for testing [31] (Table 1).

### 2.3. Model Calibration and Evaluation

We used correlative models and the relationship between occurrence data and environmental variables to predict potential distribution based on ecological niche models (ENM). We calibrated candidate models in Maxent 3.4.1 [32], and model selection was achieved using the kuenm R package [33]. We assessed all potential combinations of linear (l), quadratic (q), product (p), threshold (t), and hinge (h) feature types, in tandem with 9 regularization multiplier values (0.1, 0.3, 0.5, 0.7, 1, 3, 5, 7 and 10), and the 26, 247, and 120 environmental data sets described above, for *L. agraria*, *L. ingenua*, and *L. sativae*, respectively. Therefore, we explored 1170 candidate models for *L. agraria*, 15,561 for *L. ingenua*, and 5400 for *L. sativae* (Table 1). We evaluated the significance, performance, and complexity of each candidate model to choose the optimal parameter settings, as follows. Significance testing was via partial receiver operating characteristic (pROC) tests [34]; values of partial ROC were calculated based on a maximum acceptable omission error rate of *E* = 0.05. Omission rates were determined using a random 50% of the occurrence data, and model predictions were binarized via a modified least training presence thresholding approach (*E* = 0.05). Finally, we evaluated model complexity using the Akaike information criterion with correction for small sample size (AICc), following Warren and Seifert (2011) [35]. All modeling processes were included in the kuenm R package [33].

**Figure 1:**
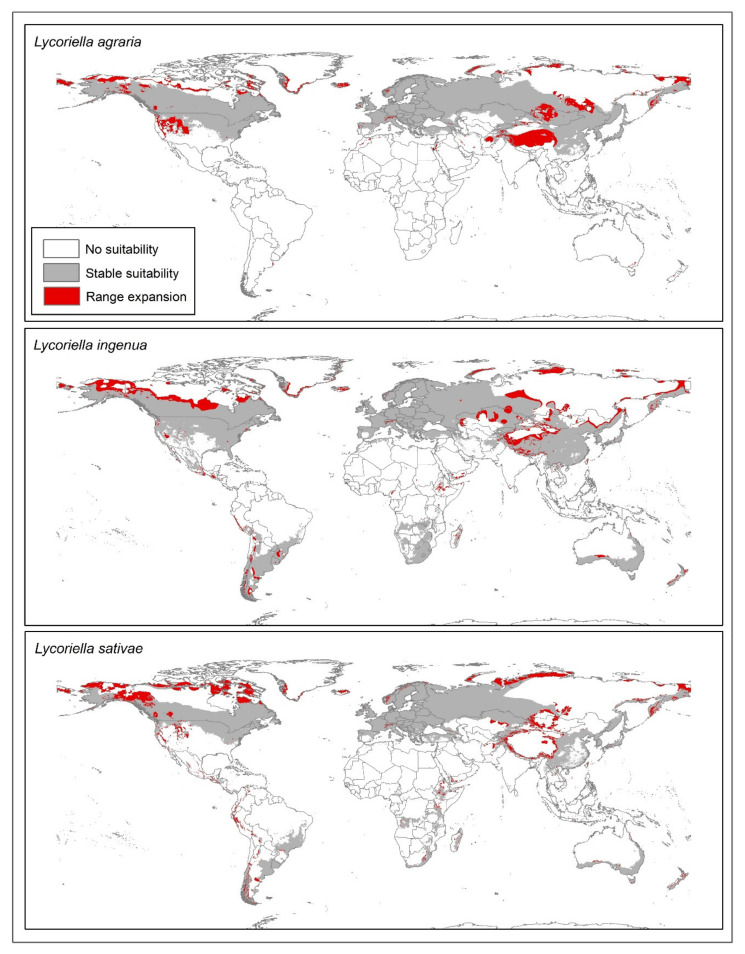
Potential distribution of three *Lycoriella* species under present and future climate conditions under two emissions scenarios (RCP 4.5 and RCP 8.5). Models show potential for range expansion worldwide in areas with low extrapolation risk.

We used a hypothesis of the accessible area (**M**) for each species to calibrate our models [36,37], using buffers of 50 km around occurrence data points that remained after spatial thinning. Final models were taken as the median of the 10 replicates for the best models and were projected worldwide. Model summaries were generated from thresholded median model projections (Figure 1) using the *E* = 0.05 value. We used the kuenm package [33] for these final steps as well. For each future-climate scenario (RCP 4.5 and RCP 8.5), we transferred the models and evaluated extrapolation conditions through MOP analysis [38], using the ntbox R package [39].

We summarized the projections of the models as medians of the replicate models using a modified least presence threshold value of *E* = 0.05. Binary maps for future conditions were used to determine uncertainty in terms of disagreement among predictions from the different GCMs (Figure 1) and median summarize for both scenarios (Figures S4–S6). We summed the maps and used the overlap between the present and future potential distribution areas to determine the prediction stability and range increase for each species in geographic areas with low extrapolation risk based on MOP analysis (Figures S7–S9).

## 3. Results

We created and evaluated 22,131 candidate models for the three *Lycoriella* species at the worldwide level, (Table 1). For *L. agraria*, of 1170 candidate models, 669 were significant (*p* < 0.05) and 575 had omission rates below 5%; of the significant, low-omission models, 7 were selected according to low complexity (AICc; Table 1). Of 15,561 candidate models for *L. ingenua*, 6898 were significant and 6789 models had omission rates below 5%; we selected 6 models based on complexity. Finally, we generated 5400 candidate models for *L. sativae*, of which 1323 were significant and 1061 had omission rates below 5%. We selected 7 models according to AIC criteria (Table 1).

Nine variables were identified as key in our ENMs (Table 2). In general, *Lycoriella* species showed relationships with seasonality in temperature and precipitation, and with variables related to cold temperatures and wet seasons (Table 2), with variable contributions ranging from 4.6 to 49.8%. The maximum number of variables for best models was in *L. sativae*, including large differences in variable contribution (Table 2).

**Figure 2:**
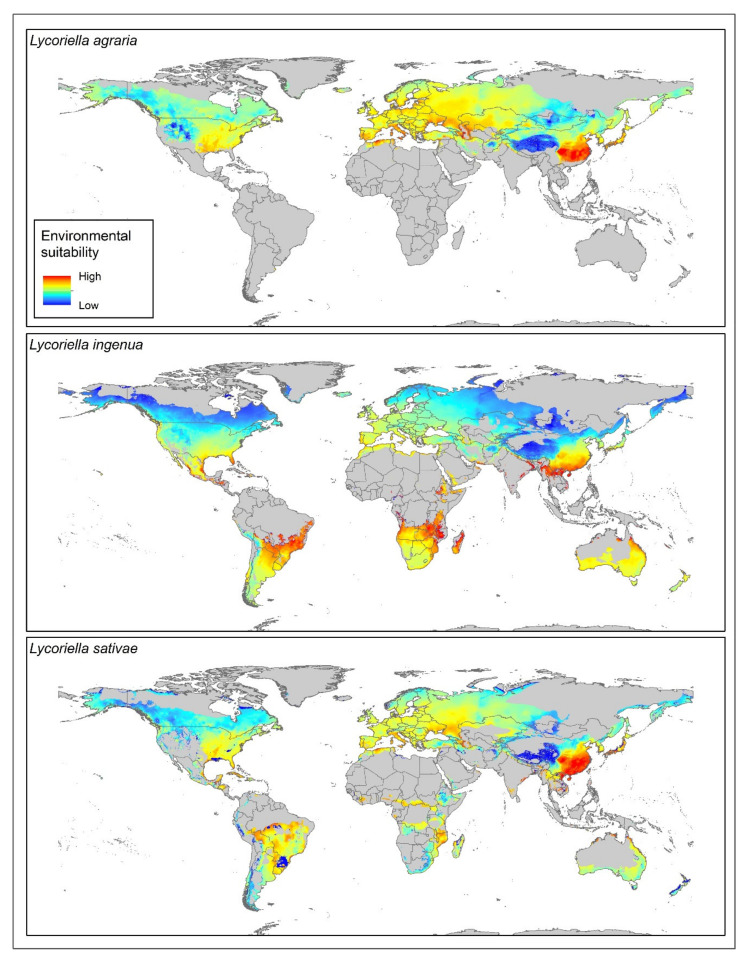
Environmental suitability for three *Lycoriella* species under current climate conditions worldwide.

Currently, suitable areas for *Lycoriella* species include much of the Northern Hemisphere, except for parts of Greenland, Russia, and northern China. Several areas in the Southern Hemisphere would also be suitable for *L. ingenua* and *L. sativae*: South America, southern Africa, and Australia (Figure 1 and Figure 2). The model for *L. agraria* indicated high suitability in parts of North America, except Mexico (Figure 1 and Figure 2), as well as much of Eurasia except for Russia, the Indian Subcontinent, and Southeast Asia. Much of the Americas were indicated as suitable areas for *L. ingenua*, except for parts of Canada, Alaska, Central America, and northern South America. *Lycoriella sativae* showed high suitability in the Americas, except in the western United States, northern Canada, central Mexico, and parts of South America (e.g., northern Brazil, Pacific Coast). Eastern and southern Asia were not suitable for this species, nor were much of Australia, North Africa, or parts of central and southern Africa. 

Stable, suitable conditions for the three *Lycoriella* species were the dominant pattern in comparisons of current and future potential distributions (Figure 1, Figures S4–S6). Potential range expansion for the three species was noted in North America and Southeast Asia (Figure 1, Figures S4–S6). Range reductions were detected in each species but were covered (less than ~78,000 km^2^) in disaggregated pixels; however, the main areas of reduction were in Asia (southern China and Mongolia). The broadest range expansions for *L*. *agraria* were anticipated in Asia (China, Russia, and Mongolia). In contrast, for *L*. *ingenua*, our results did not show a homogeneous pattern of potential range expansion; however, we noted increases in suitability in the Americas, Africa, Asia, Europe, and Australia. The biggest changes in the potential distribution of *L*. *sativae* were in North America and western parts of South America (Figure 1). New potential range areas were also identified in Alaska and Canada (Figure 1). A potential overlap in the range of *Lycoriella agraria* and *L. sativae* was indicated in the western United States (Nevada, Arizona, Idaho, Wyoming, and Colorado) (Figure 1 and Figure 2). Potential range overlaps for *L. agraria* with *L. ingenua*, and *L. ingenua* with *L. sativae* were noted in central and western China (Qinghai, Xizang, and Xinjiang), central Kazakhstan, northern and northwestern Mongolia, northern Siberia, and the border regions between China and Mongolia (Figure 1 and Figure 2).

## 4. Discussion

It is generally accepted that environmental changes will modify species’ geographic distributions worldwide [40]. Understanding how these changes will influence species’ distributions is particularly key for economically important species. Sciaridae species occur almost worldwide [10], and include important pests of mushroom crops [3], mainly in the genera *Bradysia* and *Lycoriella* [6].

*Lycoriella* includes the most threatening pests (e.g., our three species), and causes major damage to mushroom production [4]. In Korea, the most economically critical oyster mushroom pest among the six mushroom fly species is *L. ingenua* [11]. Usually, *L. sativae* is the most abundant in fields, but it is much less damaging than *L. ingenua* in mushroom culture [3].

How climate change will affect the geographic distributions of economically important sciarid species remains an open question. According to Sawangproh et al. [41], ambient temperature can affect not only the survival and larval development of sciarid flies, but also their feeding activity. As such, damage in mushroom crops or nurseries will be influenced by lower or higher temperatures. Apart from regional species checklists, little is known about the factors that drive these species’ distributions, so consequently little is known about the impacts of climate change on the future distributions of these species. These insects are easily transported by human activities, and once they reach a suitable environment, they can build up their population, which can lead to major economic losses and establish populations in mushroom production areas. 

Few studies have investigated the presence of sciarids in the Afrotropical region. Chidziya et al. [42] assessed *L. ingenua* (as *L. mali*) to be the most damaging mushroom fly in Zimbabwe, but provided no occurrence records for the species. Katumanyane et al. [43] reported for the first time the presence of both *L. ingenua* and *L. sativae* in South Africa. Our model predicted suitable environmental conditions for these species in the southern portion of the African continent, including the above-mentioned countries (Figure 1 and Figure 2), though no points from either country were included in the dataset used in model calibration.

The dominant and most serious pest species in mushroom crops in North America is *L. ingenua* [12]. Our results show that for the USA, for example, the current environmental suitability for this species is moderate for the entire West Coast and most of the southeastern part of the country, including most of the East Coast (Figure 1, Figure S5). Most of California presents high environmental suitability for the species, which is particularly relevant because California ranks second in the number of mushroom growers in the country, after Pennsylvania [9].

Pennsylvania itself currently has moderate environmental suitability (Figure 2), and our model predicts stable environmental suitability for the state under future scenarios (Figure S5). These results should be taken into consideration, since they could lead to major economic losses to mushroom producers, considering that about 66% of all US mushroom growers are located in this state [9].

In South America, on the other hand, mushroom production is still incipient. It plays a growing social role as it provides a different source of income for producers at the local level. Brazil is the most outstanding case in South America, although efforts to cultivate mushrooms are beginning in other countries [44].

So far, no official record of species of *Lycoriella* exists for Brazil. Our model showed high environmental suitability in most of southern and southwestern Brazil for *L. ingenua* and *L. sativae* (Figure 2). As such, once these species are introduced into the country, they will likely have the ability to establish stable populations, a fact that must be regarded with care because most Brazilian mushroom production is concentrated in the southern and southwestern states. The introduction of *Lycoriella* species to the country would pose an extra threat to Brazilian mushroom growers, who already face problems with other sciarid and scatopsid species [45,46].

The genus *Lycoriella* significantly reduces mushroom production inside greenhouses; these species also may impact other agricultural species (e.g., strawberry, nursery plants [6,47,48]. Our results show areas around the world with suitable conditions for these flies (Figure 2). We are particularly concerned about greenhouse availability, although we did not incorporate possible competition with other species into our models. However, *Lycoriella* species show very broad ecological niches with high invasive potential, from Brazil to Alaska. We suggest that experimental physiological studies that address the fundamental niche of these species more directly will be an important next step in protecting food production in greenhouses, and to characterize areas with environmental conditions that characterize the physiological limits of the development of *Lycoriella* populations.

## Figures and Tables

**Table 1:** Best models selected and evaluated based on statistical significance (partial ROC), performance (omission rates: OR), and complexity (AICc). This model was calibrated and projected using the environmental variables shown in Table 2.

*Lycoriella* Species	Mean AUC Ratio	pROC *p* Value	Omission Rate at 5%	AICc	Delta AICc	Reg. Multiplier	Feature Classes
*L. agraria* 1170 models	1.000	0	0.04	829.260	0.000	1	lqpt
	1.049	0	0.04	830.493	1.232	1	lqpt
	1.000	0	0	830.664	1.401	3	lqpth
	1.000	0	0	830.667	1.407	3	lqpth
	1.000	0	0	830.667	1.407	3	lqpth
	1.000	0	0.04	831.205	1.945	1	lqpt
	1.000	00	0.04	831.208	1.948	1	lqpt
*L. ingenua* 15,561 models	1.036	0	0.01	2425.36	0	3	l
	1.035	0	0.03	2425.366	0.005	0.1	l
	1.036	0	0.03	2425.366	0.005	0.3	l
	1.036	0	0.03	2425.366	0.005	0.5	l
	1.035	0	0.03	2425.366	0.005	0.7	l
	1.035	0	0.03	2425.366	0.005	1	l
*L. sativae* 5400 models	1.052	0	0.031	2766.137	0	3	l
	1.047	0	0.046	2766.874	0.736	0.1	l
	1.044	0	0.046	2766.874	0.736	0.3	l
	1.046	0	0.046	2766.874	0.736	0.5	l
	1.045	0	0.031	2766.874	0.736	0.7	l
	1.043	0	0.015	2766.874	0.736	1	l
	1.000	0	0	2767.922	1.784	3	pth

**Table 2:** Models and variables that were relatively uncorrelated (Pearson’s correlation ≤ 0.8) for *Lycoriella* species. The models were built and tested used 26 variables sets for *L. agraria*, 247 variables sets for *L. ingenua*, and 120 variables sets for *L. sativae*.

Species	Uncorrelated Variables	Variable Contribution (%)
*L. agraria*	Mean diurnal range	4.60
	Mean temperature of warmest quarter	48.67
	Mean temperature of coldest quarter	0.00
	Precipitation of wettest quarter	22.67
	Precipitation of driest quarter	24.05
*L. ingenua*	Temperature seasonality	28.90
	Maximum temperature of warmest month	0.00
	Mean temperature of coldest quarter	49.80
	Precipitation of wettest quarter	21.30
*L. sativae*	Mean diurnal range	38.26
	Maximum temperature of warmest month	29.44
	Temperature annual range	0.00
	Mean temperature of coldest quarter	7.89
	Annual precipitation	8.18
	Precipitation of wettest quarter	5.77
	Precipitation of driest quarter	10.41

## Data Availability

The data presented in this study are available in Supplementary Materials.

## Supplementary Materials

The following are available online at https://www.mdpi.com/article/10.3390/insects12090831/s1, Figure S1: Known distribution areas of Lycoriella agraria, Figure S2: Known distribution areas of Lycoriella ingenua, Figure S3: Known distribution areas of Lycoriella sativae, Figure S4: Projections of environmental suitability for Lycoriella agraria in current and future scenarios, Figure S5: Projections of environmental suitability for Lycoriella ingenua in current and future scenarios, Figure S6: Projections of environmental suitability for Lycoriella sativae in current and future scenarios, Figure S7: MOP analysis of extrapolation risk from the calibration area under projection current and future conditions for Lycoriella agraria, Figure S8: MOP analysis of extrapolation risk from the calibration area under projection current and future conditions for Lycoriella ingenua, Figure S9: MOP analysis of extrapolation risk from the calibration area under projection current and future conditions for Lycoriella sativae, Table S1: General circulation models used in ecological niche model projections in RCP 4.5 and RCP 8.5 for 2050, Table S2; Bibliographic references used for occurrences data of Lycoriella species.
