# Supplementary material for: Effects of Climatic Change on the Potential Distribution of Lycoriella Species (Diptera: Sciaridae) of Economic Importance
Source: Insects. 2021 Sep 15;12(9):831. doi: 10.3390/insects12090831 (PMC8471568; doi:10.3390/insects12090831)

Table S1. General circulation models used in ecological niche model projections in RCP 4.5 and RCP 8.5 for 2050.

General circulation model acronym	Institution
bnu_esm	Beijing Normal University Earth System Model
cesm1_bcg	National Center for Atmospheric Research – The University of Arizona
cesm1_cam5	National Center for Atmospheric Research – The University of Arizona – NSF-DOE-NCAR
csiro_access1_3	Commonwealth Scientific and Industrial Research Organization (CSIRO) and Bureau of Meteorology (BOM), Australia
csiro_access1	Commonwealth Scientific and Industrial Research Organization (CSIRO) and Bureau of Meteorology (BOM), Australia
gfdl_cm3	NOAA Geophysical Fluid Dynamics Laboratory
gfdl_esm2g	NOAA Geophysical Fluid Dynamics Laboratory
gfdl_esm2m	NOAA Geophysical Fluid Dynamics Laboratory
giss_e2_r	NASA Goddard Institute for Space Studies USA
giss_e2_h	NASA Goddard Institute for Space Studies USA
inm_cm4	Russian Institute for Numerical Mathematics
miroc_esm	University of Tokyo, National Institute for Environmental Studies, and Japan Agency for Marine-Earth Science and Technology
miroc_esm_chem	University of Tokyo, National Institute for Environmental Studies, and Japan Agency for Marine-Earth Science and Technology
miroc_miroc5	University of Tokyo, National Institute for Environmental Studies, and Japan Agency for Marine-Earth Science and Technology

mohc_hadgem2_cc	UK Met Office Hadley Centre
mohc_hadgem2_es	UK Met Office Hadley Centre
mri_cgcm3	Meteorological Research Institute
ncar_ccsm4	US National Centre for Atmospheric Research
ncc_noresm1_m	Norwegian Climate Centre
nimr_hadgem2_ao	UK Met Office Hadley Centre
cccma_canesm2	Canadian Centre for Climate Modelling and Analysis Canada
mpi_esm_lr	Max Planck Institute for Meteorology, Germany

Table S2. Bibliographic references used for occurrences data of *Lycoriella* species.

Lycoriella agraria

Broadley, A., Kauschke, E., & Mohrig, W. (2018). Black fungus gnats (Diptera: Sciaridae) found in association with cultivated plants and mushrooms in Australia, with notes on cosmopolitan pest species and biosecurity interceptions. *Zootaxa*, *4415*(2), 201-242.

El Ouazzani, N. E. H., Heller, K., & Kettani, K. (2019, May). The first checklist of black fungus gnats (Diptera: Sciaridae) of Morocco. In *Annales de la Société entomologique de France (NS)* (Vol. 55, No. 3, pp. 274-290). Taylor & Francis.

Komarov, S. S. (2009). A review of species of the sciarid-fly genus *Lycoriella* Frey (Diptera, Sciaridae) of the Altai fauna. *Entomological Review*, *89*(2), 175-180.

Menzel, F., Smith, J. E., & Chandler, P. J. (2006). The sciarid fauna of the British Isles (Diptera: Sciaridae), including descriptions of six new species. *Zoological Journal of the Linnean Society*, *146*(1), 1-147.

Mohrig, W., Heller, K., Hippa, H., Vilkamaa, P., & Menzel, F. (2012). Revision of the black fungus gnats (Diptera: Sciaridae) of North America. *Studia dipterologica*, *19*(1-2), 141-286.

Roháček, J., & Ševčík, J. (2009). Diptera of the Poľana Protected Landscape Area-Biosphere Reserve (Central Slovakia). *SNC SR, Administration of the PLA–BR Pol'ana, Zvolen.. 323p.*

Lycoriella ingenua

Andreadis, S. S., Cloonan, K. R., Bellicanta, G. S., Paley, K., Pecchia, J., & Jenkins, N. E. (2016). Efficacy of Beauveria bassiana formulations against the fungus gnat *Lycoriella ingenua*. *Biological Control*, *103*, 165-171.

Arimoto, M., Uesugi, R., Hinomoto, N., Sueyoshi, M., & Yoshimatsu, S. I. (2018). Molecular marker to identify the fungus gnat, *Bradysia* sp.(Diptera: Sciaridae), a new pest of Welsh onion and carrot in Japan. *Applied entomology and zoology*, *53*(3), 419-424.

Broadley, A., Kauschke, E., & Mohrig, W. (2018). Black fungus gnats (Diptera: Sciaridae) found in association with cultivated plants and mushrooms in Australia, with notes on cosmopolitan pest species and biosecurity interceptions. *Zootaxa*, *4415*(2), 201-242.

Chidziya, E., Mutangadura, D., Jere, J., & Siziba, L. (2013). A comparative evaluation of locally available substrates for rearing and studying biology of sciarid fly, *Lycoriella mali*. *Academia Journal of Biotechnology*, *1*(4), 057-061.

Jess, S., & Bingham, J. F. W. (2004). The spectral specific responses of *Lycoriella ingenua* and *Megaselia halterata* during mushroom cultivation. *The Journal of Agricultural Science*, *142*(4), 421-430.

Lewandowski, M., Sznyk, A., & Bednarek, A. (2004). Biology and morphometry of *Lycoriella ingenua* (Diptera: Sciaridae). *Biological Letters*, *41*(1), 41-50.

Marín-Cruz, V. H., Cibrián-Tovar, D., Méndez-Montiel, J. T., Pérez-Vera, O. A., Cadena-Meneses, J. A., Huerta, H., ... & Cruz-Rodríguez, J. A. (2015). Biología de

Lycoriella ingenua y *Bradysia impatiens* (Diptera: Sciaridae). *Madera y bosques*, *21*(1), 113-128.
Menzel, F., Smith, J. E., & Chandler, P. J. (2006). The sciarid fauna of the British Isles (Diptera: Sciaridae), including descriptions of six new species. *Zoological Journal of the Linnean Society*, *146*(1), 1-147.
Mohrig, W., Heller, K., Hippa, H., Vilkamaa, P., & Menzel, F. (2012). Revision of the black fungus gnats (Diptera: Sciaridae) of North America. *Studia dipterologica*, *19*(1-2), 141-286.
Nickle, W. R., & Cantelo, W. W. (1991). Control of a mushroom-infesting fly, *Lycoriella mali*, with *Steinernema feltiae*. *Journal of Nematology*, *23*(1), 145.
Park, I. K., Kim, J. N., Lee, Y. S., Lee, S. G., Ahn, Y. J., & Shin, S. C. (2008). Toxicity of plant essential oils and their components against *Lycoriella ingenua* (Diptera: Sciaridae). *Journal of Economic Entomology*, *101*(1), 139-144.
Park, J. M., You, Y. H., Park, J. H., Kim, H. H., Ghim, S. Y., & Back, C. G. (2017). Cutaneous microflora from geographically isolated groups of *Bradysia agrestis*, an insect vector of diverse plant pathogens. *Mycobiology*, *45*(3), 160-171.
Szlendak, E., & Lewandowski, M. (2009). Development and reproductive capacity of the predatory mite *Parasitus consanguineus* (Acari: Parasitidae) reared on the larval stages of *Megaselia halterata* and *Lycoriella ingenua*. *Experimental and Applied Acarology*, *47*(4), 285-292.
Ye, L., Leng, R., Huang, J., Qu, C., & Wu, H. (2017). Review of three black fungus gnat species (Diptera: Sciaridae) from greenhouses in China: Three greenhouse sciarids from China. *Journal of Asia-Pacific Entomology*, *20*(1), 179-184.
Yi, J. H., Park, I. K., Choi, K. S., Shin, S. C., & Ahn, Y. J. (2008). Toxicity of medicinal plant extracts to *Lycoriella ingenua* (Diptera: Sciaridae) and *Coboldia fuscipes* (Diptera: Scatopsidae). *Journal of Asia-Pacific Entomology*, *11*(4), 221-223.

Lycoriella sativae

Broadley, A., Kauschke, E., & Mohrig, W. (2018). Black fungus gnats (Diptera: Sciaridae) found in association with cultivated plants and mushrooms in Australia, with notes on cosmopolitan pest species and biosecurity interceptions. *Zootaxa*, *4415*(2), 201-242.
El Ouazzani, N. E. H., Heller, K., & Kettani, K. (2019, May). The first checklist of black fungus gnats (Diptera: Sciaridae) of Morocco. In *Annales de la Société entomologique de France (NS)* (Vol. 55, No. 3, pp. 274-290). Taylor & Francis.
Katumanyane, A., Kanzi, A. M., & Malan, A. R. (2020). Sciarid pests (Diptera: Sciaridae) from undercover crop production in South Africa. *South African Journal of Science*, *116*(3-4), 1-6.
Komarov, S. S. (2009). A review of species of the sciarid-fly genus *Lycoriella* Frey (Diptera, Sciaridae) of the Altai fauna. *Entomological Review*, *89*(2), 175-180.
Latibari, M. H., Moravvej, G., Heller, K., Rulik, B., & Namaghi, H. S. (2015). New records of Black Fungus Gnats (Diptera: Sciaridae) from Iran, including the reinstatement of Bradysia cellarum Frey. *Studia dipterologica*, *22*(1), 39-44.

Menzel, F., & Müller, W. A. (2010). Trauermücken (Diptera: Sciaridae) aus Pfahlwurzeln von Rumex obtusifolius Linnaeus in Südwest-Thüringen (Deutschland), mit Bemerkungen zur Variabilität von *Bradysia scabricornis* Tuomikoski. *Studia dipterologica*, *17*(1/2).

Menzel, F., Gammelmo, Ø., Olsen, K. M., & Köhler, A. (2020). The Black Fungus Gnats (Diptera, Sciaridae) of Norway–Part I: species records published until December 2019, with an updated checklist. *ZooKeys*, *957*, 17.

Menzel, F., Vilkamaa, P., & Smith, J. E. (2013). Overview of the Black Fungus Gnats from the Tristan da Cunha archipelago, including a redescription of *Hyperlasion viridiventris* (Frey) (Diptera: Sciaroidea: Sciaridae). *Beiträge zur Entomologie-Contributions to Entomology*,*63*(2), 283-296.

Mohrig, W., Kauschke, E., & Broadley, A. (2019). Revision of black fungus gnat species (Diptera, Sciaridae) described from the Hawaiian Islands by DE Hardy and WA Steffan, and a contribution to the knowledge of the sciarid fauna of the Galápagos Islands. *Zootaxa*, *4590*(4), 401-439.

Navarro, M. J., Escudero, A., Gea, F. J., López-Lorrio, A., García-Morrás, J. A., & Ferragut, F. (2000). Determinación y abundancia estacional de las poblaciones de dípteros (Diptera: Phoridae y Sciaridae) en los cultivos de champiñón en Castilla-La Mancha. *Boletín de Sanidad Vegetal Plagas*, *4*, 527-536.

Rudzinski, H. G., & Baumjohann, K. (2009). Neue Trauermücken aus Spanien (Diptera: Sciaridae). *Entomologische Zeitschrift*, *119*(5), 211-218.

Shin, S. G., Lee, H. S., & Lee, S. (2012). Dark winged fungus gnats (Diptera: Sciaridae) collected from shiitake mushroom in Korea. *Journal of Asia-Pacific Entomology*, *5*(1), 174-181.

Figure S1. Known distribution areas of *Lycoriella agraria*. Occurrences used for calibration and evaluation of the model. Below is shown the accessible area (**M**): 50 km buffer around to all occurrences.

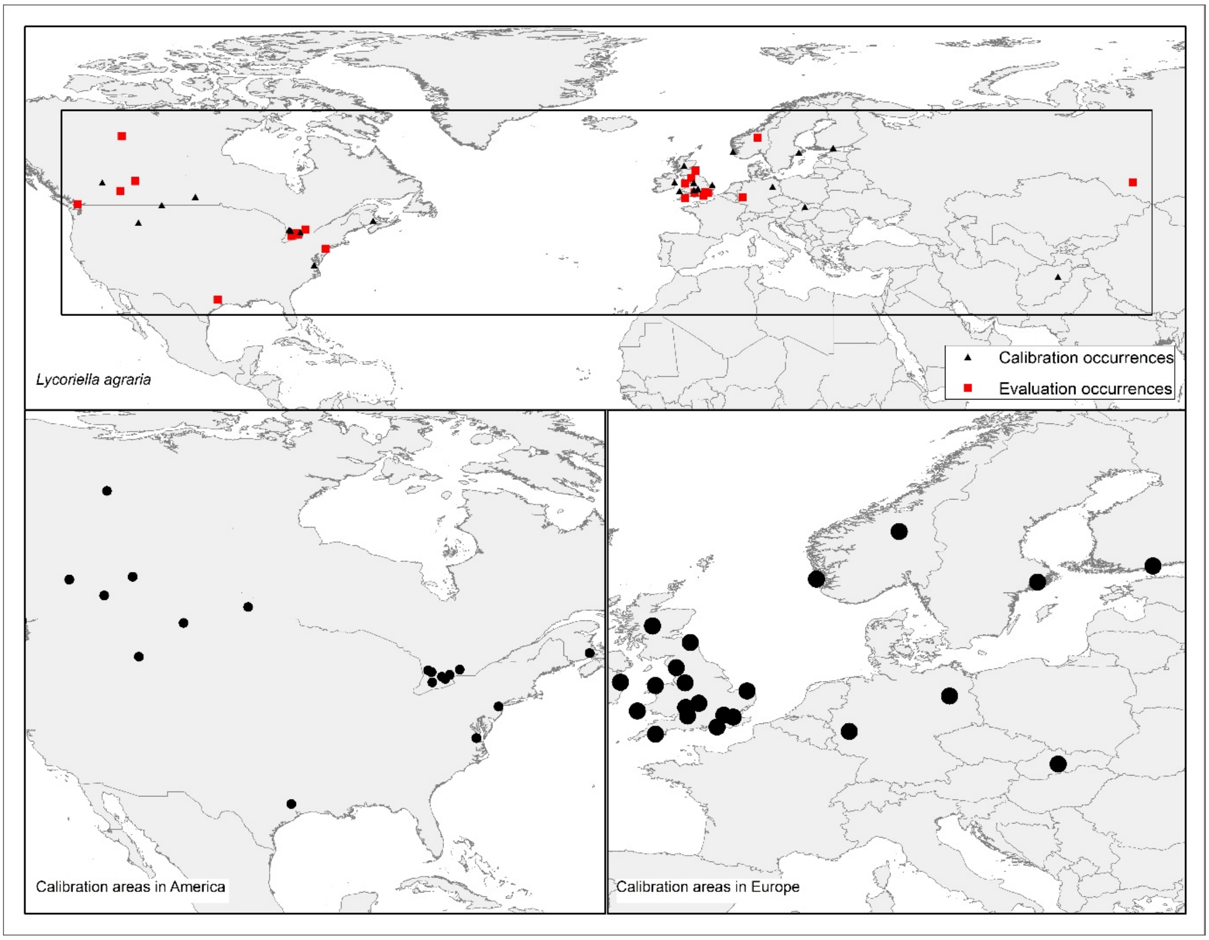

Figure S2. Known distribution areas of *Lycoriella ingenua*: Occurrences used for calibration and evaluation of the model. Below is shown the accessible area (**M**): 50 km buffer around to all occurrences.

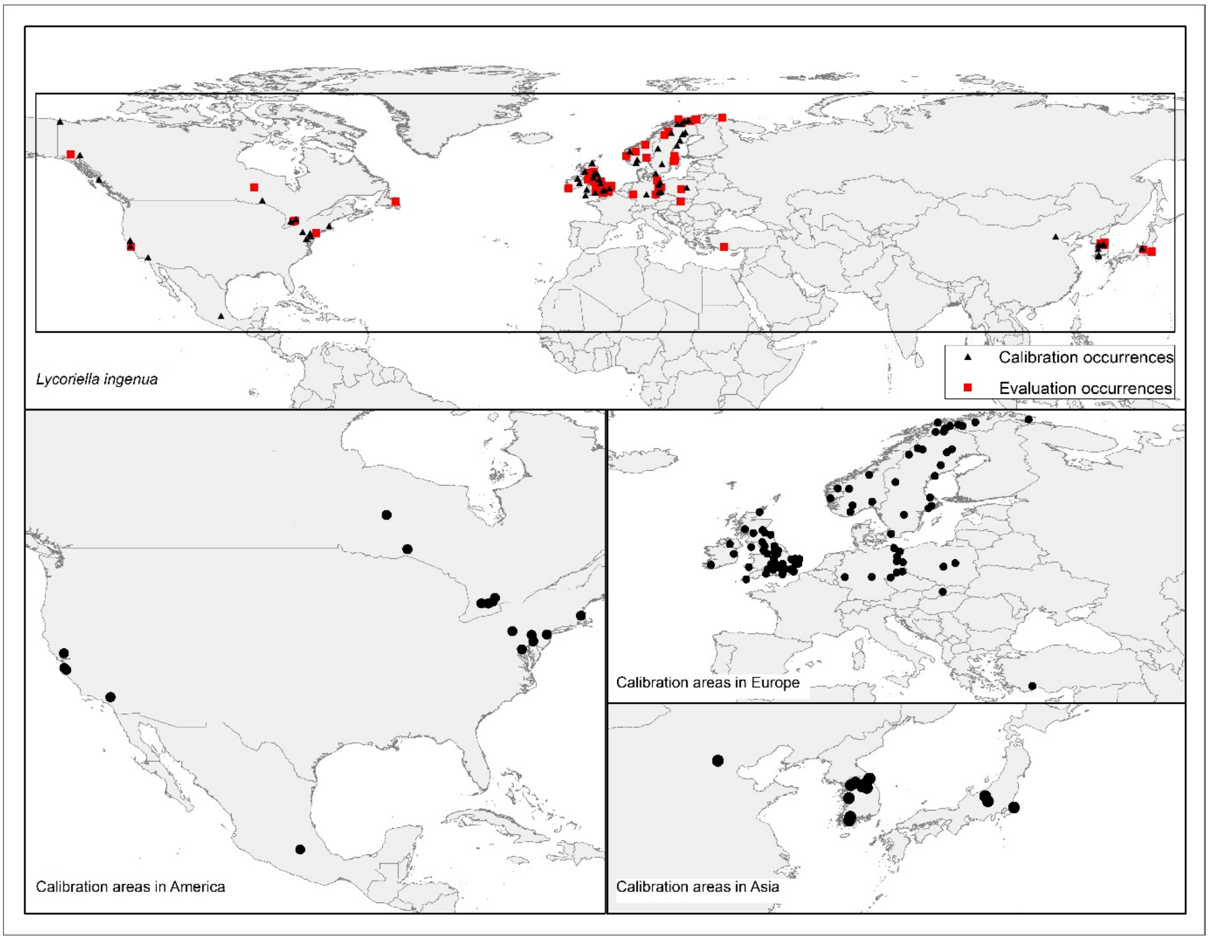

Figure S3. Known distribution areas of *Lycoriella sativae*: Occurrences used for calibration and evaluation of the model. Below is shown the accessible area (**M**): 50 km buffer around to all occurrences.

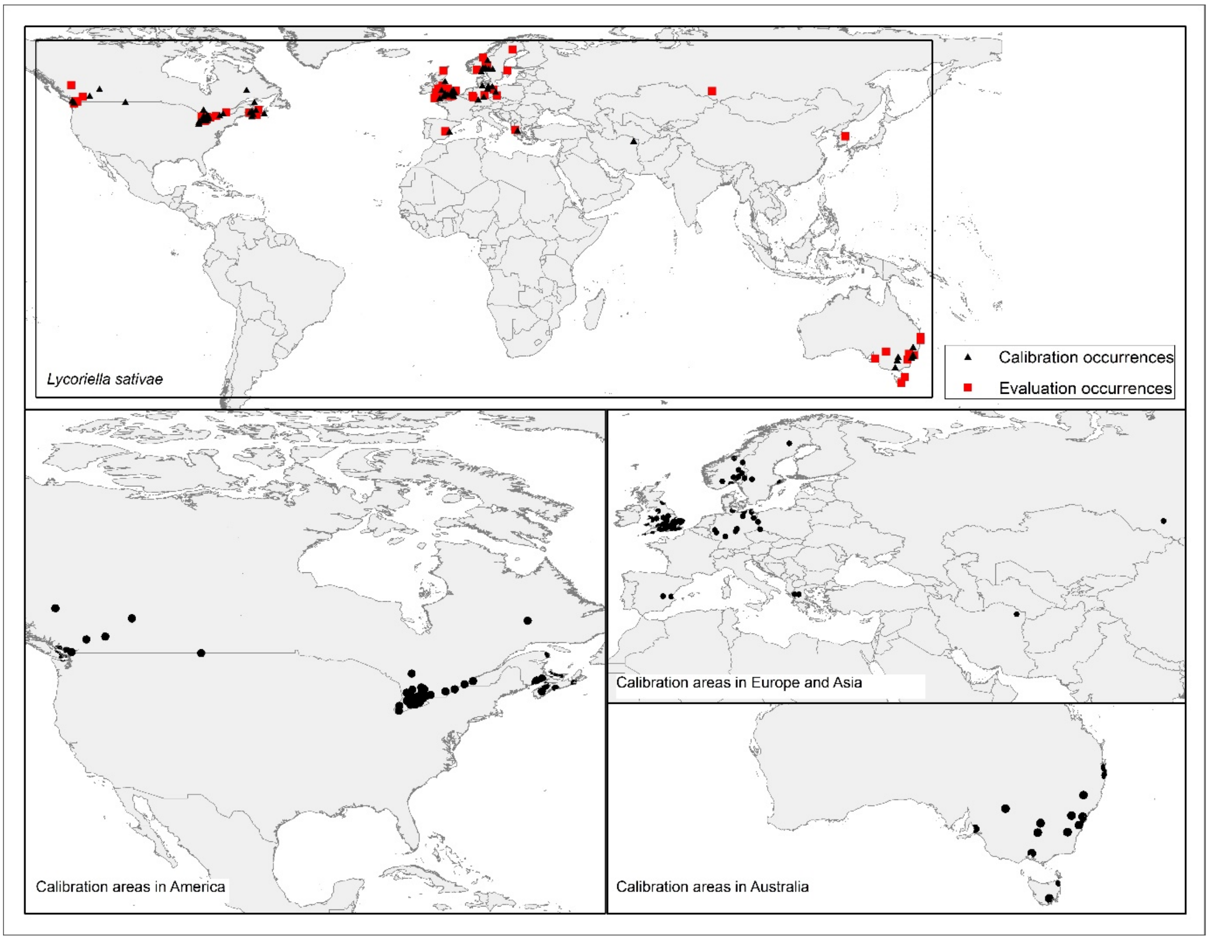

Figure S4. Projections of environmental suitability for *Lycoriella agraria* in current and future scenarios (RCP 4.5 and RCP 8.5) for 2050 in low extrapolation risk areas.

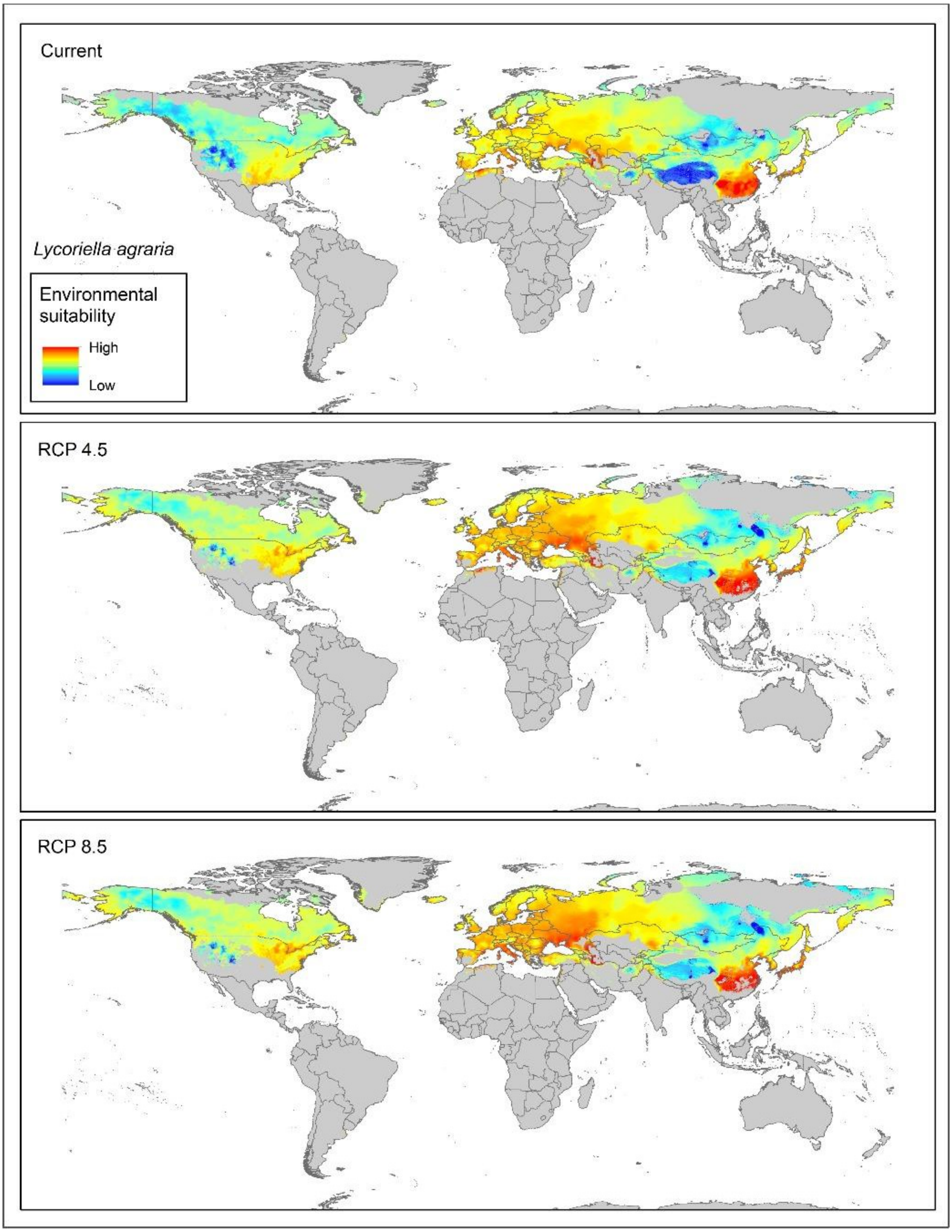

Figure S5. Projections of environmental suitability for *Lycoriella ingenua* in current and future scenarios (RCP 4.5 and RCP 8.5) for 2050 in low extrapolation risk areas.

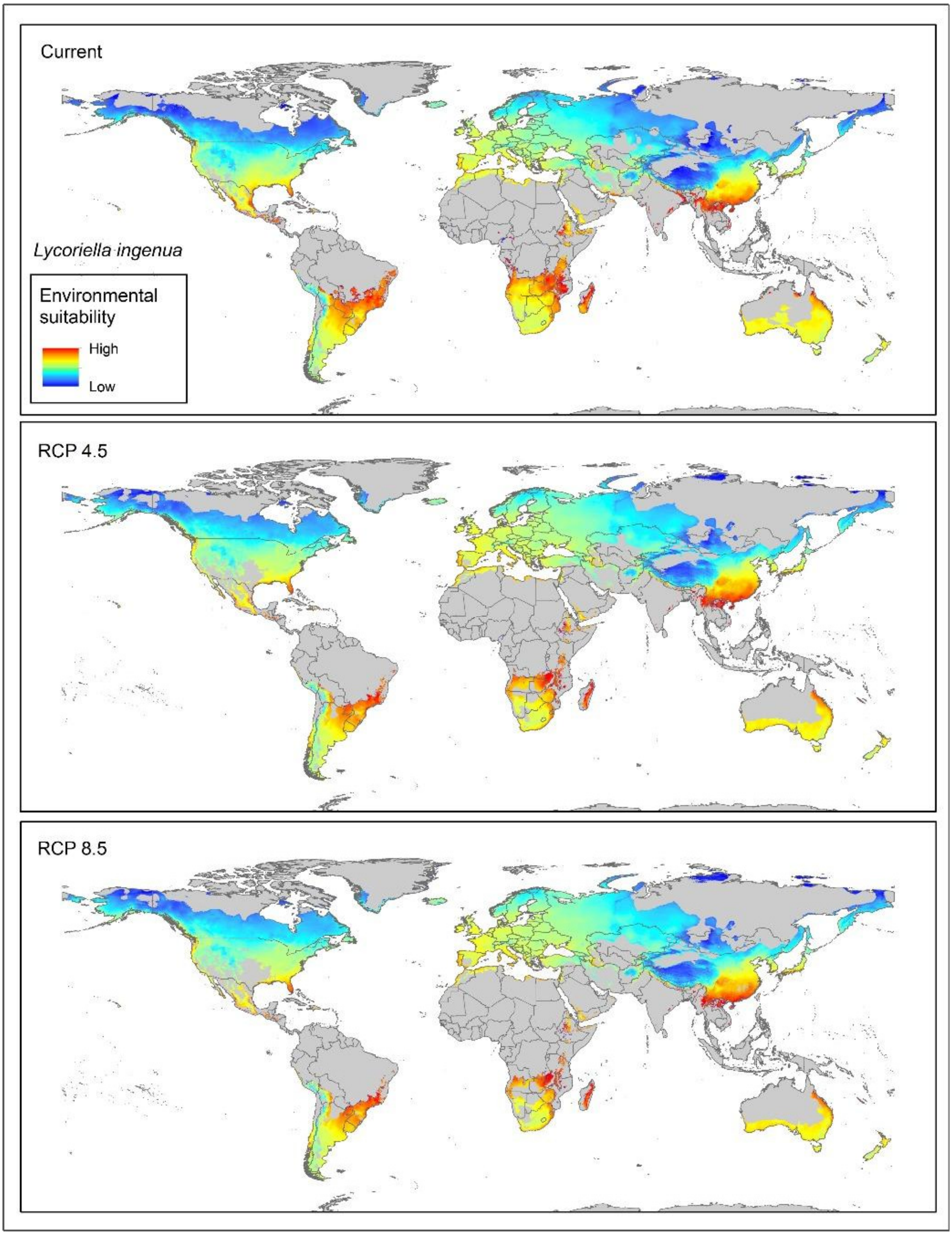

Figure S6. Projections of environmental suitability for *Lycoriella sativae* in current and future scenarios (RCP 4.5 and RCP 8.5) for 2050 in low extrapolation risk areas.

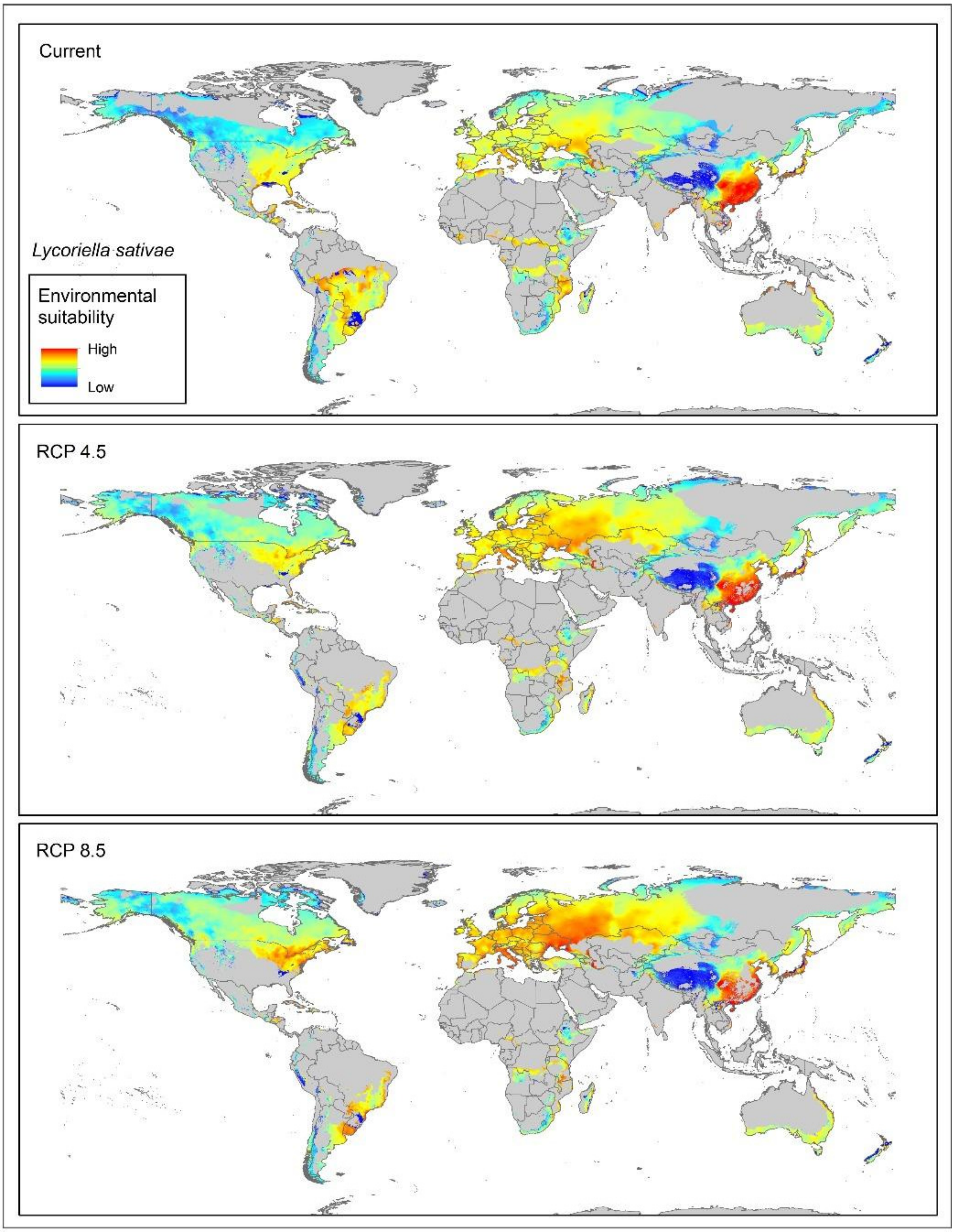

Figure S7. MOP analysis of extrapolation risk from the calibration area under projection current and future conditions for *Lycoriella agraria*. Red areas represent strict extrapolation. Blue areas represent similarity between the calibration area (M) and the RCPs scenarios of projections.

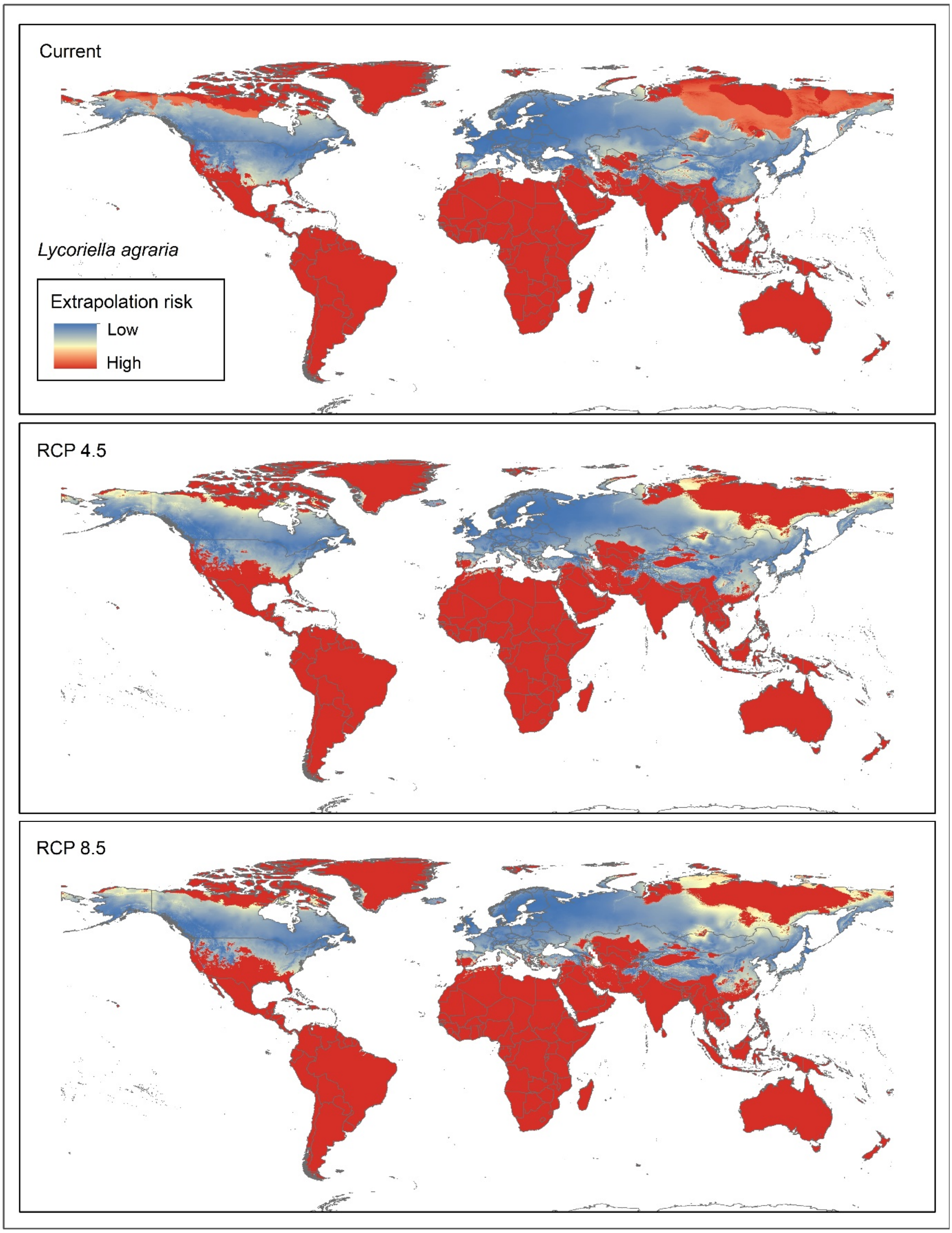

Figure S8. MOP analysis of extrapolation risk from the calibration area under projection current and future conditions for *Lycoriella ingenua*. Red areas represent strict extrapolation. Blue areas represent similarity between the calibration area (M) and the RCPs scenarios of projections.

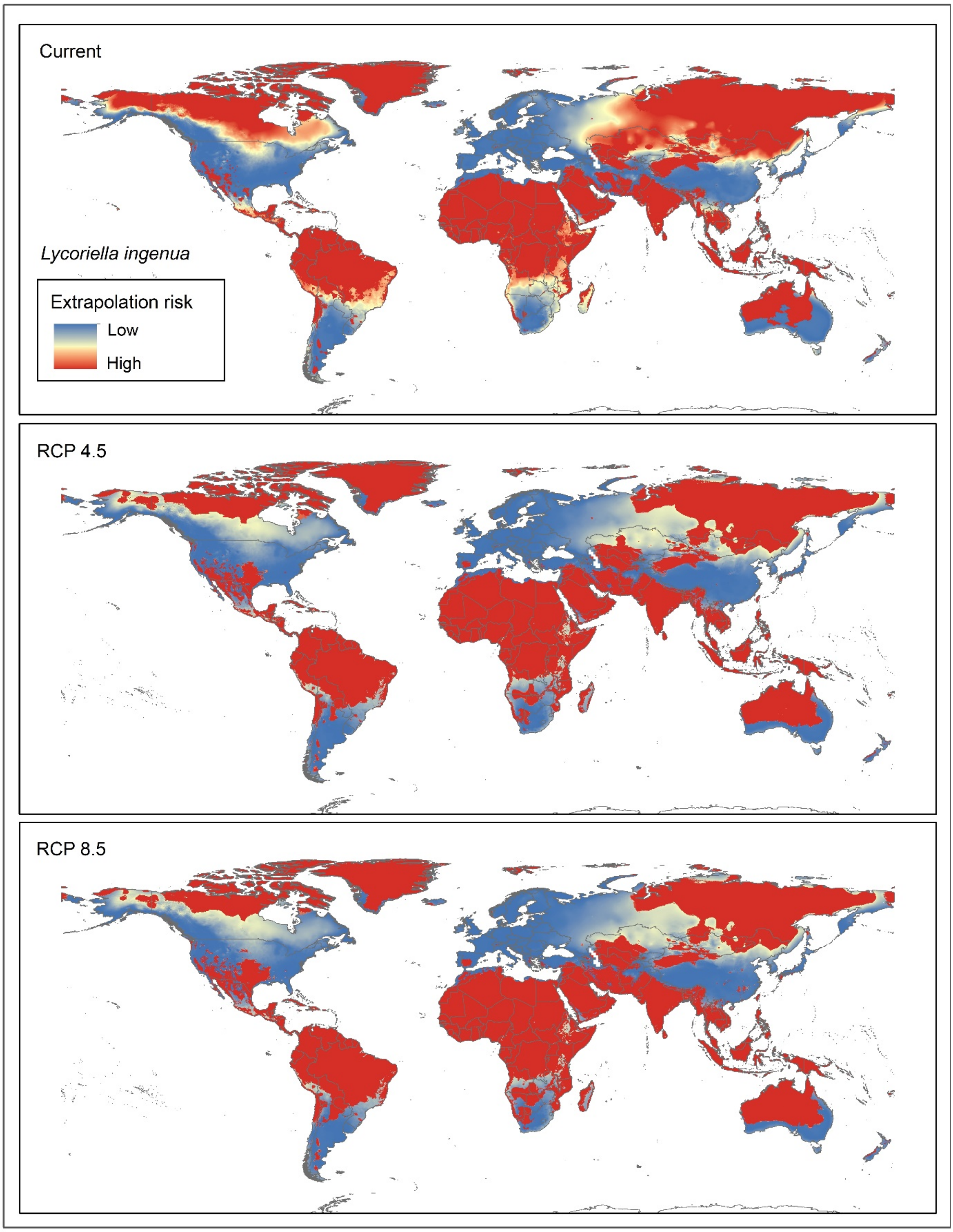

Figure S9. MOP analysis of extrapolation risk from the calibration area under projection current and future conditions for *Lycoriella sativae*. Red areas represent strict extrapolation. Blue areas represent similarity between the calibration area (M) and the RCPs scenarios of projections.

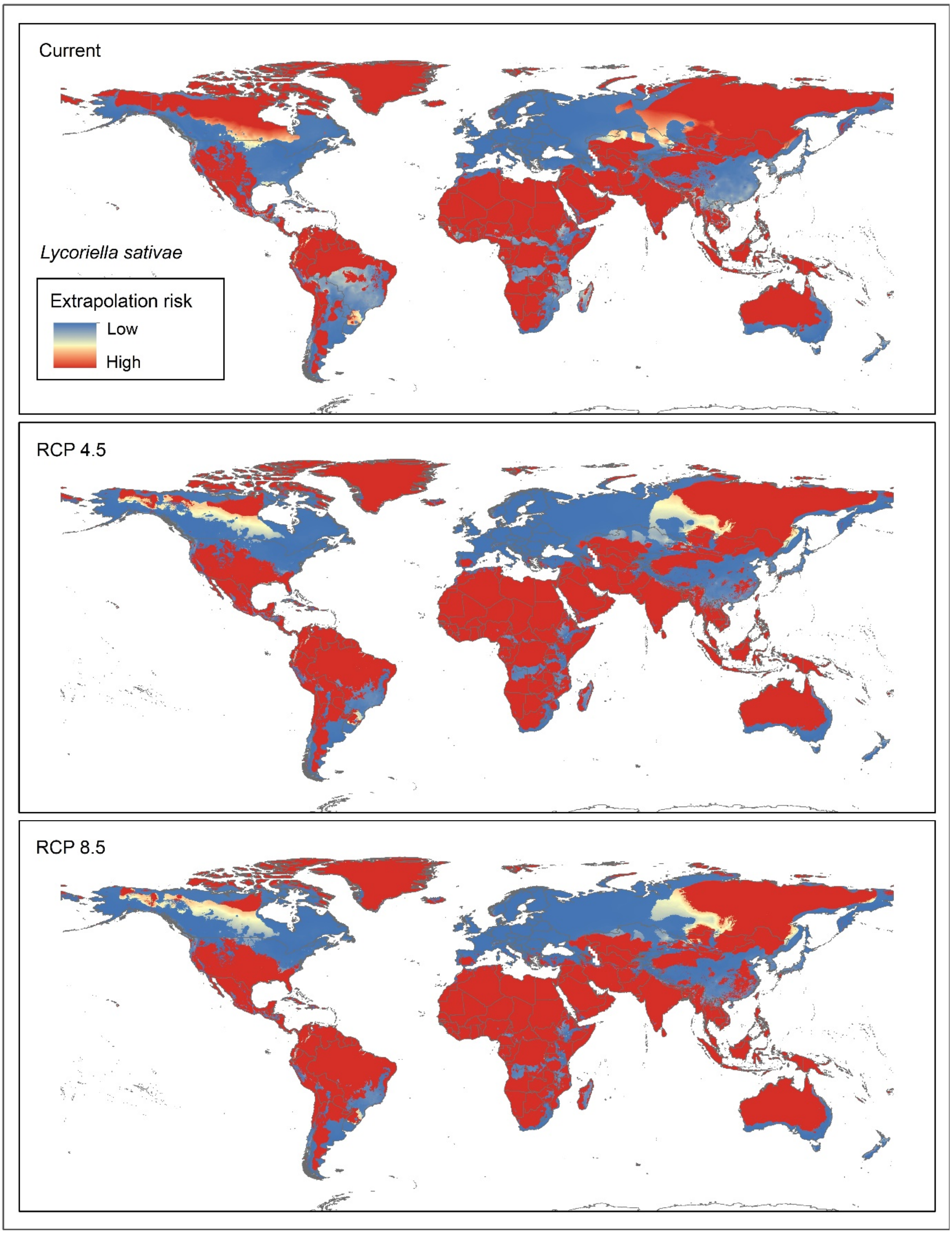

Supplement: Supplementary file 1 [file insects-12-00831-s001.zip › insects-1313012-supplementary.pdf]
